# Current evidence of the economic value of One Health initiatives: A systematic literature review

**DOI:** 10.1016/j.onehlt.2024.100755

**Published:** 2024-05-09

**Authors:** Aashima Auplish, Eleanor Raj, Yoeri Booijink, Katinka de Balogh, Marisa Peyre, Katrin Taylor, Keith Sumption, Barbara Häsler

**Affiliations:** a Food and Agriculture Organization of the United Nations (FAO), 00153 Rome, Italy; b Centre de Coopération Internationale en Recherche Agronomique Pour le Développement (CIRAD), Montpellier Cedex 5 34398, France; c Royal Veterinary College, London NW1 0TU, UK

**Keywords:** One Health, Economic evaluation, Return on investment, Systematic review, Added value

## Abstract

Funding and financing for One Health initiatives at country level remain challenging as investments commonly require demonstrated evidence of economic value or returns. The objectives of this review were to i) identify, critically analyse and summarise quantitative evidence of the net economic value of One Health initiatives; ii) document methodologies commonly used in the scientific literature; and iii) describe common challenges and any evidence gaps. Scientific databases were searched for published literature following the PRISMA guidelines and an online survey and workshop with subject matter experts were used to identify relevant grey literature. Studies were included if they reported on quantitative costs and benefits (monetary and non-monetary) and were measured across at least two sectors. Relevant publications were analysed and plotted against the six action tracks of the Quadripartite One Health Joint Plan of Action to help classify the initiatives. Ninety-seven studies were included. Eighty studies involved only two sectors and 78 reported a positive economic value or return. Of those studies that reported a positive return, 49 did not compare with a sectoral counterfactual, 28 studies demonstrated an added value of using a cross-sectoral approach, and 6 studies demonstrated an added value of One Health communication, collaboration, coordination, and capacity building. Included studies most frequently related to endemic zoonotic, neglected tropical and vector-borne diseases, followed by health of the environment and food safety. However, diversity in economic analysis methodology between studies included resulted in difficulty to compare or combine findings. While there is a growing body of evidence of the value of One Health initiatives, a substantial part of the evidence still focuses on “traditional” One Health topics, particularly zoonoses. Developing a standardised and practical approach for One Health economic evaluation will facilitate assessment of the added value and gather evidence for One Health to be invested in and endorsed by multiple sectors.

## 1 Introduction

One Health is an approach that recognises that the health of humans, domestic and wild animals and the wider environment are closely linked and interdependent. It mobilises multiple sectors, disciplines, and communities and aims to strengthen health systems to prevent disease threats to health and ecosystems, while addressing the collective need for clean water, energy and air, safe and nutritious food, taking action on climate change, and contributing to sustainable development [1]. One Health is rooted in effective communication, collaboration, coordination, and capacity building (hereinafter known as ‘the four Cs of One Health’), between people from different sectors, backgrounds and disciplines in society [1] and is gaining momentum as a practical approach at the local, national, and global levels. Conversely, Planetary Health encompasses a broader view that includes the interactions between human civilization, ecosystems, and the Earth's natural systems [2]. Both approaches, however, recognise the vital importance of understanding and addressing these complex interactions to promote improved well-being and health of the planet.

 Recently, the Quadripartite for One Health, i.e., the Food and Agriculture Organization of the United Nations (FAO), United Nations Environment Programme (UNEP), World Health Organization (WHO), and World Organization for Animal Health (WOAH) developed the One Health Joint Plan of Action (JPA) (2022–2026), to support the mainstreaming and implementation of One Health globally [3]. The One Health JPA is built around six interdependent action tracks that collectively contribute to achieving sustainable health and food systems, reduced global health threats and improved ecosystem management and provide a framework for prioritising One Health action that requires investment.

Strengthening systems and coordination across sectors through leveraging greater investments are expected to provide a positive economic value or return on investment (ROI) as well as reduced costs, disease risks and indirect socioeconomic losses [4]. Economic evaluations conducted systematically across sectors determine whether there is a return on the One Health investment and inform and justify resource allocation decisions for the required expenditure or scaled investment. These can serve as an advocacy tool, assisting policymakers in understanding how costs and benefits are generated and shared across sectors. Moreover, it provides information on the generation of private and public goods; another important consideration when it comes to One Health investments.

Reviews conducted to date on the added value of the One Health approach have focused on existing evaluation frameworks, metrics or outcomes used for One Health evaluations. Falzon et al. [5] assessed studies that describe a monetary and non-monetary quantitative outcome when using a One Health approach and found that the majority of the studies reported positive economic outcomes with rabies, malaria and air pollution being the most common health issues addressed. Baum et al. [6] reviewed the current status of One Health frameworks and case studies reporting One Health metrics and found a lack of quantitative metrics demonstrating the expected benefits of a One Health approach. However, both reviews did not explicitly focus on demonstrating the added value of investments in One Health initiatives. Similarly, Häsler et al. [7] reviewed One Health benefits and found that while there were many benefits described qualitatively, there was a dearth of studies reporting quantitative outcomes and a lack of a standardised approach to estimate these benefits. Further, investment costs were not considered explicitly in this review. Naylor et al. [8] described quantitative evaluation methods for interventions related to cross-sectoral issues and proposed an approach for evaluating such interventions. Because none of the existing reviews on One Health looked explicitly at quantitative measures for the ROI of One Health initiatives, there is an important gap in evidence for those who need to make decisions on investment in One Health or alternative actions. Therefore, a systematic literature review was conducted to provide an overview of existing evidence on the economic value of One Health initiatives presented against the six action tracks of the One Health JPA.

The objectives of this review were to i) identify, critically analyse and summarise the existing published and unpublished quantitative evidence of the economic value of One Health initiatives (monetary and non-monetary); ii) document methodologies commonly used in the scientific literature to obtain these values, iii) describe the common challenges associated with One Health economic evaluations and any evidence gaps. The outcomes of this review can support decision making for efficient allocation of resources towards One Health initiatives.

## 2 Methods

### 2.1 Overview

This systematic review followed the Preferred Reporting Items for Systematic Reviews and Meta-Analyses (PRISMA) guidelines [9].

The outcome of interest was the economic value of One Health initiatives (monetary and non-monetary) expressed in quantitative figures. No restrictions were defined in terms of methods used in the publications reviewed, as long as there was a comparison between the costs (investment) of a One Health initiative and their benefits or effects compared to a counterfactual. This could include, for example, financial methods such as the ROI (defined as an economic metric used to understand the profitability or efficiency of an investment and compares the net benefit or loss and the initial cost of the investment), or cost-effectiveness and cost-benefit analyses that allow comparing wider societal monetary and non-monetary benefits to the costs.

Any health-related program or project that was integrated or interdisciplinary and involved at least two of the human health, animal health, wildlife health, and environment health (including plant health) sectors were considered [10]. A cross-sectoral measure of either costs or health outcomes was required. The One Health initiative should ideally have had a cross-sectoral or multi-sectoral aspect in communication, collaboration, coordination or capacity building. Publications reviewed were plotted against the six action tracks of the One Health JPA to classify the One Health initiatives included in this review.

### 2.2 Search strategy

Studies were identified through Pubmed, Web of Science and Scopus using a title, abstract and keyword search without any restrictions in terms of language or year. Searches were run on 4 August 2022. The search strategy comprised terms representing two concepts: One Health (including referring to the six action tracks of the One Health JPA), and economic evaluation. The search terms used were selected to build on existing evidence generated from previously published literature reviews [[5], [6], [7], [8],^11^] and to present evidence that relates directly to the One Health JPA [3]. No restrictions on the date of publication were applied for the search. The detailed search strategies for the databases and sources consulted are reported in Table S1, supplementary materials.

The search results were downloaded into Endnote reference manager, where duplicates were removed and prepared for screening with random allocation to reviewers. Additionally, published and unpublished studies identified through an online international survey (conducted in parallel to this review) and virtual workshop with 35 One Health economics experts were included for review. Subject matter experts were invited to submit any relevant publications, which were then subjected to the same reviewing process outlined. Further information about the online survey and workshop can be found in Table S2, supplementary materials.

### 2.3 Screening and selection criteria

Double blind screening was carried out in Rayyan [12]. Four reviewers (AA, BH, ER, YB) independently assessed each reference allocated to them using the inclusion and exclusion criteria presented in Table 1. Articles were also excluded where there was no abstract or full text available electronically. Any conflicts between decisions to include or exclude were handled by one of the reviewers who had not already screened the study in question. All articles that met the inclusion criteria were subjected to full text screening, which was conducted by one of the reviewers. Double full-article screening was conducted if there were doubts around whether the article should be included. For review articles, reference lists were checked for additional articles not found during the search and primary articles were subjected to the same screening process. Only studies in English were included due to limited availability of resources.

**Table 1:** Inclusion and exclusion criteria.

Inclusion criteria	Exclusion criteria
One Health: Studies that assess One Health initiatives (involving health aspects in two or more sectors with cross-sectoral or multi-sectoral aspects in communication, collaboration, coordination or capacity building) OR One Health: Studies of initiatives that measure health outcomes (monetary or non-monetary) in another sector or population	Only (uni-)sectoral evaluation studies: studies which have activities and outcomes only in one sector and/or perform evaluation of an initiative only involving one sector
Economic evaluation studies that compare the investments (costs) and return (monetary and non-monetary benefits)	Evaluation studies with no comparison between investment (cost) and return (monetary and non-monetary benefits), e.g., disease impact or burden only studies
Quantitative costs or outcomes measured in one or more sectors	Quantitative costs or outcomes not measured
Peer-reviewed articles or other publications (such as policy briefs or project reports) that fulfilled criteria above and listed the aim, methods, and results.	Any publications that would not describe the aim, methods and results (such as editorials, commentary articles and letters to the editor).

### 2.4 Data extraction and analysis

Data from the articles included after the full text screening were extracted into an Excel file and information categorised including the (1) year of publication, (2) publication type, (3) publication format, (4) type of One Health initiative, (5) One Health JPA action track to which the initiative primarily relates (6) value added or not from a One Health approach, (7) reporting cross-sectoral communication, collaboration, coordination and capacity building (one of the four Cs of One Health) [1], (8) scale of implementation, (9) geographic location of initiative, (10) sectors involved, (11) type of economic evaluation, (12) type of return, (13) metrics used for quantitative measures in sectors involved, (14) types of non-monetary benefits (if included), (15) key recommendations related to the ROI of the One Health initiative, and (16) challenges or gaps of the evaluation.

For all analyses, standard economic evaluation criteria were used to categorise the studies into those with a positive or negative economic value. For example, studies reporting a positive net present value; cost saved per disability-adjusted life year (DALY) averted; benefit cost ratio (BCR) >1.0 were classified as having a positive economic value. Data related to challenges in conducting economic analyses of One Health initiatives were examined using thematic analysis [13] and are summarised in Table 6.

Finally, the studies were categorised by evidence of One Health integration: i) “Strong evidence of One Health integration” included studies that measured explicitly the added value of at least one of the four Cs of One Health; ii) “Moderate evidence of One Health integration” encompassed studies that measured the added value of One Health comparing a cross-sectoral approach to a sectoral counter-factual; and iii) “Weak evidence of One Health integration” covered studies that measured cross-sectoral effects, but did not compare the One Health initiative to a sectoral counterfactual.

## 3 Results

### 3.1 Flowchart

**Fig. 1:**
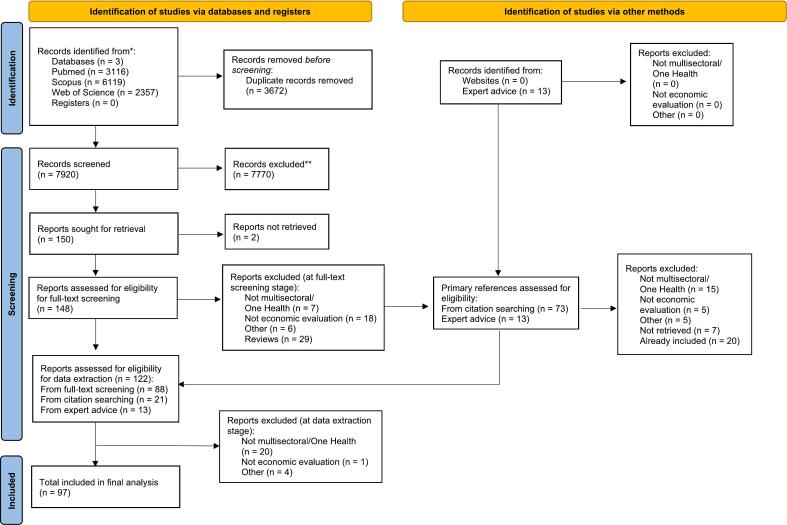
PRISMA flow diagram.

Figure 1 presents the PRISMA flow diagram of the systematic literature review; a total of 97 studies were included in the final analysis.

### 3.2 Descriptive results

Included studies were published between 1990 and 2022. Most were scientific journal articles (91.8%, *n* = 89), followed by reports (6.3%, *n* = 6), book chapters (both 1.0%, *n* = 1) and policy briefs (1.0%, n = 1). The majority of studies used a combination of empirical evidence and modelling (38.1%, *n* = 37) or modelling only (36.1%, *n* = 35).

Ninety seven percent of studies involved human or public health (*n* = 94); 73% involved animal health (*n* = 71); 35% involved environment health (including plant health) (*n* = 34) and 16% involved wildlife health (*n* = 16). Other industries or sectors involved in studies included the food industry, education, transport sector and energy industry. Most studies involved only two sectors (83%, *n* = 80), 13% involved three sectors (*n* = 13) and 4% involved four sectors (*n* = 4). The One Health JPA action track (hereinafter referred to as ‘action track(s)’) that the included studies primarily related to is presented in Table 2. The most frequent was action track 3 (42%, *n* = 41).

**Table 2:** One Health JPA action track to which the One Health initiative of studies included relates to (*n* = 97).

One Health JPA Action Track	n (%)
Action track 1: Enhancing One Health capacities to strengthen health systems	2 (2.1)
Action track 2: Reducing the risks from emerging and re-emerging zoonotic epidemics and pandemics	7 (7.2)
Action track 3: Controlling and eliminating endemic zoonotic, neglected tropical and vector-borne diseases	41 (42.3)
Action track 4: Strengthening the assessment, management and communication of food safety risks	21 (21.6)
Action track 5: Curbing the silent pandemic of AMR	2 (2.1)
Action track 6: Integrating the environment into One Health	24 (24.7)

With reference to geographic distribution of One Health initiatives, 29% of studies were conducted in Europe (*n* = 28); 20% in Asia (*n* = 19); 16% in Africa (n = 16); 16% in North America (*n* = 16); 3% in Oceania (n = 3) and 2% in South America (*n* = 2). Eight percent of studies (*n* = 8) evaluated initiatives on a global scale and 5% did not reference a geographic location (*n* = 5). In total, 57 different countries were referenced. In terms of the scale of the initiative, 30% of studies (*n* = 29) focused on initiatives conducted at a local level, 14% in multiple states or provinces (*n* = 14); 40% at a national level (*n* = 39), 3% at a regional level (n = 3) and 6% at an international level or global level (*n* = 6). Six percent of studies (n = 6) did not report the scale of the initiative.

The most common method used was cost-benefit analysis (CBA) (35.0%, *n* = 34), followed by cost-effectiveness analysis (CEA) (28%, *n* = 27) and cost-utility analysis (5.0%, *n* = 5). Twenty-seven studies (28%) listed monetised costs and benefits but did not appear to follow a specific methodology for economic evaluation. Common metrics used to describe monetary outcomes were benefit-cost ratio (19% *n* = 18), cost savings (18%, *n* = 17), net benefits (13%, *n* = 13) and average or incremental cost-effectiveness ratio (11%, *n* = 11 respectively). The median time horizon for studies included within the final analysis was 10 years (minimum: 1 year, maximum: 150 years, IQR: 15 years). In terms of perspective of the economic evaluation, 45% of studies used a societal perspective and 29% used a public health perspective.

### 3.3 Positive and negative economic values of One Health initiatives

Overall, 80 % of all included studies (*n* = 78) reported a positive economic value or return on investment while 10% (*n* = 10) of studies reported a negative economic value and 9% (*n* = 9) of studies did not demonstrate clear evidence of either a positive or negative economic value or return on investment. Table 3 shows the grouping of studies by evidence of One Health integration – most studies fell into the “weak evidence” group that measured cross-sectoral effects without the use of a sectoral counterfactual.

**Table 3:** Evidence of One Health integration in the 97 studies reviewed.

Evidence of One Health integration	Total number of studies (%)	Number of studies with positive economic return (%)
Strong evidence: added value of the four Cs of One Health (communication, collaboration, coordination or capacity building) [1]	12 (12%)	6 (50%)
Moderate evidence: added value of cross-sectoral approach compared to a sectoral counterfactual	31 (32%)	28 (90%)
Weak evidence: cross-sectoral initiative where approach not compared with sectoral counterfactual	66 (68%)	49 (74%)

#### 3.3.1 Added value of the four Cs of One Health (strong evidence of One Health integration)

**Fig. 2:**
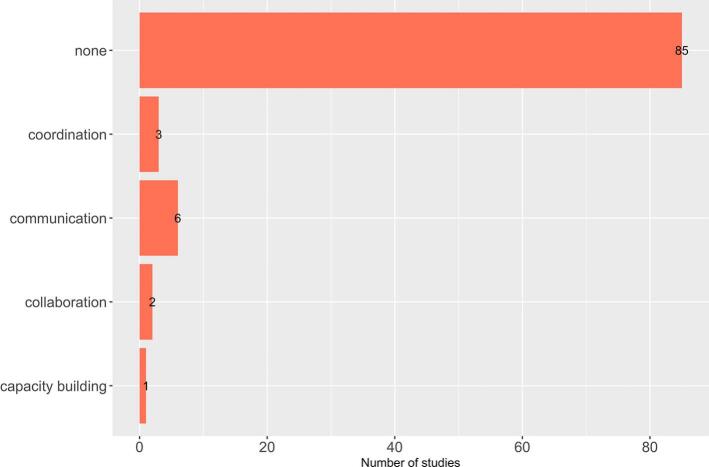
Number of included studies with evidence of at least one of the four C's of One Health across sectors (*n* = 97).

Twelve studies out of 97 (12%) estimated added value through one or more of the four C's of One Health (Fig. 2). Seven of these compared a cross-sectoral initiative with a sectoral approach thereby estimating the added value of the four C's of One Health. Six of these seven studies showed a positive net economic value; all of them related to the action track 3 (Table 4). The majority evaluated initiatives for the control of zoonotic disease, in particular, for canine-mediated rabies.

**Table 4:** One Health initiatives demonstrating positive economic evidence of the added value of the four C's across sectors (“strong evidence of One Health integration”).

One Health initiative and level of implementation	Four Cs of One Health demonstrated by sectors involved	Economic evaluation method	Type and quantification of economic value; counterfactual for evaluation
Conducting One Health epidemiological study of Rift Valley fever in South Africa implemented at local level [14]	**Collaboration** between human health, agricultural/veterinary services	Costs and benefits listed and monetised (ex-post)	Cost savings through shared resource use between sectors of 35% or US $6432.61 throughout the program, compared with no resource sharing
Post-exposure prophylaxis and canine mass vaccination against rabies in Chad implemented at local level [15]	**Communication** between human health, agricultural/veterinary services	CEA (ex-post)	US $63 per DALY averted over 20 years for scenario involving human post-exposure prophylaxis (PEP), mass dog vaccination and maximal communication between human health and veterinary workers (One Health communication), compared with PEP alone and PEP and dog vaccination (no One Health communication)
Novel integrated bite case management (IBCM) program for the control of human rabies in Haiti implemented at local level [16]	**Communication** between human health, agricultural/veterinary services	CEA (ex-post)	With IBCM (involving communication and information exchange between human health and animal health sector) US $3534–$7171 per human rabies case averted, compared with no IBCM over two years
IBCM program for the control of human rabies as part of a pilot zoonoses and emerging infectious disease prevention and control program in Indonesia implemented at local level [17]	**Communication** (**collaboration**, **coordination**, **capacity building**) between the human health, agricultural/veterinary services, environment/wildlife services	CBA/CEA (ex-post)	Under low disease transmission scenario, the BCR for the IBCM program (involving communication and information exchange between human health and animal health sector) over the last three years estimated at 6.56, with US $2124 per DALY averted, compared with not using IBCM. Under high disease transmission scenario, the BCR estimated at 14.35 with US $972 per DALY averted
Oral rabies vaccination program for control of domestic dog-coyote rabies in USA implemented in multiple provinces/states [18]	**Communication** between human health, agricultural/veterinary services	CBA (ex-post)	BCR of 3.38–13.12 over 13 years (of program) compared with using uni-sectoral approach of human PEP alone
One Health integrated surveillance system for West Nile virus (WNV) to mitigate risk of transmission via blood transfusion implemented at local (state) level [19]	**Communication** between human health, agricultural/veterinary services	Costs and benefits listed and monetised (ex-post)	€160,921 in averted costs of potential human cases of WNV neuroinvasive disease associated with infected blood transfusion during seven-year period, compared with a uni-sectoral approach

#### 3.3.2 Evidence of economic value of a cross-sectoral initiative (moderate evidence of One Health integration)

Thirty one of the 97 studies (32%) conducted an evaluation where a cross-sectoral initiative was compared with a sectoral initiative. Twenty nine percent of these studies (*n* = 28) demonstrated a positive economic value (Table 5). Action track 3 was most represented in these studies (*n* = 25). The majority (*n* = 20) of these studies focused on the control of human disease through the application of disease control measures in the animal reservoir. There were no studies related to action tracks 1 and 5 and only one study each related to action tracks 2, 4 and 6 respectively.

**Table 5:** Studies demonstrating positive economic value of a cross-sectoral approach (“moderate evidence of One Health integration”).

One Health initiative and level of implementation	Sectors involved in the initiative	One Health JPA action track⁎ addressed	Economic evaluation method	Type and quantification of positive economic value. For all studies the counterfactual for evaluation was a uni-sectoral initiative
H7N9 vaccination program in poultry in Guangxi, China implemented in multiple provinces/states [20]	Human health, agricultural/veterinary services	Action track 2	CBA (ex-post)	BCR⁎⁎ of the three-year H7N9 vaccination program was 18.6 (90 %PI: 15.4; 21.8) Total NPV⁎⁎⁎ reached CNY 1.63 billion (90 %PI: 1.37 billion; 1.89 billion)
Combining drug and environmental treatments for environmentally transmitted NTDs (using schistosomiasis as case study), in Senegal implemented at local level [21]	Human health and environment services	Action track 3	CBA (ex-ante)	Implementing environmental controls with mass drug administration (MDA) can significantly reduce the time span over which one has to administer drug treatment, resulting in about a 10% reduction in MDA expenditures.
Human health benefits from livestock vaccination for brucellosis, in Mongolia implemented at national level [22]	Human health, agricultural/veterinary services	Action track 3	CEA (ex-post)	Societal average BCR⁎⁎ of 3.2 (95% CI 2.27–4.37) over ten-year mass vaccination program
Integrated control program for Taenia solium, soil transmitted helminths (STH) and classical swine fever in northern Lao PDR implemented at local level [23]	Human health, agricultural/veterinary services	Action track 3	CEA (ex-post)	US $14 per DALY^a^ averted for all combined interventions i.e., cysticercosis in humans and pigs, STH in humans and CSF in pigs (classified as very cost-effective based on WHO cost-effectiveness thresholds) over 1.5-year program
Dog anthelmintic prophylaxis combined with a sheep and goat vaccination program for echinococcosis control in the Tibetan Plateau [24]	Human health, agricultural/veterinary services	Action track 3	CEA (ex-ante)	U.S.$106.88 per DALY^a^ averted (95% CI U.S.$88.63–127.99) with the proposed dog deworming and sheep and goat vaccination program over five-year period
Control of taeniasis and cysticercosis in humans, pigs and cattle (level of implementation not stated) [25]	Human health, agricultural/veterinary services	Action track 3	CEA (ex-ante)	ICER^b^ for meat inspection and treatment of humans who are infected with taeniasis is 234.069 and ICER^b^ for (integrated approach of) meat inspection, pigs and cattle vaccination, treatment of humans who are infected with taeniasis and improved hygiene and sanitation is 0.226 over ten-year period
Control of dog-mediated rabies by canine vaccination and animal-birth-control program in Jaipur, India implemented at local level [26]	Human health, agricultural/veterinary services	Action track 3	CBA (ex-post)	Monetary BCR⁎⁎ of 8.5; societal BCR of 58.4 and US $39.71 per DALY^a^ averted over 23 years of the program
Elimination of human rabies through an integrated One Health approach (dog vaccination; rabies education; and intensified human and animal rabies surveillance), in Goa, India implemented at local (state) level [27]	Human health, agricultural/veterinary services	Action track 3	CEA (ex-post)	US $526 per DALY^a^ averted over seven-year program
National program of dog rabies elimination in canine-rabies infected countries [28]	Human health, agricultural/veterinary services	Action track 3	CEA (ex-ante)	Total annual costs of human prevention through post-exposure treatment combined with disease control in the animal reservoir becomes less than cost of human oriented prevention alone after the 5th year of program initiation
Rabies control programs (PEP, mass vaccination of dogs, targeted sterilisation, education and development of dog managed zones in public areas) In Sri Lanka implemented at local level [29]	Human health, agricultural/veterinary services	Action track 3	Costs and benefits listed and monetised (ex-ante)	Over four years, the intervention cost US $1.03 million more than the baseline scenario in 2011 prices; 738 DALYs^a^ averted and an increase in acceptability amongst non-dog owners (5.68 increase mean acceptance score)
Canine rabies vaccination programs to prevent human rabies in rural Tanzania implemented at local level [30]	Human health, agricultural/veterinary services	Action track 3	CEA (ex-ante)	Canine vaccination in Ngorongoro is very cost- effective for annual campaigns that reach 20% to 30% coverage. In Serengeti, vaccination would be very cost-effective at coverage from 25% to 70% (based on WHO cost-effective threshold) over ten years
Canine rabies vaccination programs in East Africa implemented at local level [31]	Human health, agricultural/veterinary services	Action track 3	CEA (ex-ante)	US $460–368 cost per DALY^a^ averted or US $13–17 cost per life year gained with annual vaccination of 50% of dogs over ten years
National dog rabies prevention and control program in Mexico implemented at national level [32]	Human health, agricultural/veterinary services	Action track 3	CEA (ex-post)	US $23,000 per DALY^a^ averted, $410 per additional year-of-life, and $190 per dog rabies death averted (classified as highly cost effective based on WHO standards) over 26 years of program
Canine rabies vaccination programs in Ethiopia implemented at local level [33]	Human health, agricultural/veterinary services	Action track 3	CEA (ex-ante)	Vaccination coverages of below 50% and 70% are very cost-effective in Bishoftu and Lemunabilbilo respectively (based on WHO cost-effectiveness thresholds) over five years
Resuming disrupted canine rabies vaccination campaigns in Haiti [34]	Human health, agricultural/veterinary services	Action track 3	CEA (ex-ante)	US $1355 cost per human death averted if vaccination restarted in 2021, $1475 cost per human death averted if restarted in 2022
Canine rabies vaccination campaigns in Sub-Saharan Africa implemented at local level [35]	Human health, agricultural/veterinary services	Action track 3	CEA (ex-ante)	In Ngorongoro, biennial vaccination of 50% of the canine population had the highest ICER^b^ of $1222 per DALY^a^ averted (very cost-effective based on the WHO cost-effective threshold) and in Serengeti, biennial vaccination with a coverage of 70% had the highest ICER of $191 per DALY^a^ averted (very cost-effective based on the WHO cost-effectiveness thresholds) over ten years
Post-elimination vaccination to prevent re-establishment of dog rabies in African and Asian countries [36]	Human health, agricultural/veterinary services	Action track 3	CEA (ex-ante)	Most cost-effective to continue dog vaccination at the minimum coverage required (38% in low risk scenario, 56% in high risk scenario), with the average cost per DALY^a^ (human rabies death) averted ranging from $257 to $398 USD over a 20-year period
Mass canine rabies vaccination programs to reduce human health burden in Flores Island, Indonesia implemented at local level [37]	Human health, agricultural/veterinary services	Action track 3	CEA (ex-post)	US $4.38 per years of life lost (YLL) averted (using short action vaccine annually at 70% vaccination coverage), US $4.63 and US $2.65 per YLL averted (using long-acting vaccine annually at 50% vs 70% vaccination coverage) over 10-year campaign
Costs and benefits of rabies control in wildlife in France [38]	Human health, agricultural/veterinary services	Action track 3	CBA (ex-ante)	Cumulative annual cost of an oral vaccination strategy became beneficial in the fourth year compared to a fox depopulation strategy
Red fox oral rabies vaccination program in Ontario, Canada [39]	Human health, agricultural/veterinary services	Action track 3	CBA (ex-post)	BCR⁎⁎ of 0.49, 1.06, 1.27 or 1.36 (depending on the forecasting technique used), indicating overall program efficiency in three of four scenarios over a ten-year period
Non-typhoidal Salmonella control program in Hungary [40]	Human health, agricultural/veterinary services, food industry	Action track 4	CUA (ex-post)	ICER^b^ of €27,150 per QALY^c^ gain (below the national health technology assessment ICER threshold of 35,790 EUR per QALY gained, therefore cost-effective) over a ten-year period
Integrated health interventions (integrated vector management to control malaria, tsetse fly traps to control trypanosomiasis, push–pull technology to address stemborer and fodder shortages and improved beekeeping) in Ethiopia implemented at local level [41]	Agricultural/ veterinary services, environment services	Action track 6	Costs and benefits listed and monetised (ex-post)	Annual income over eight years from the combined interventions is 35% higher than the sum of the income gains at the household level from each intervention alone (US $368 per capita per year)

⁎ Refer to Table 2 for the list of One Health JPA action tracks.

⁎⁎ BCR: benefit-cost ratio.

⁎⁎⁎ NPV: net present value.

a DALY: disability adjusted life year.

b ICER: incremental cost-effectiveness ratio.

c QALY: quality adjusted life year.

#### 3.3.3 Economic value of studies with One Health characteristics but no evidence on the value of using a cross-sectoral vs sectoral approach (weak evidence of One Health integration)

Sixty six of the 97 studies (68%) were a health-related program or project that was integrated or interdisciplinary and involved at least two sectors but did not compare a One Health approach with a sectoral initiative as the counterfactual in the economic evaluation. Forty-nine (74%) of these studies demonstrated a positive economic return for the initiative studied. Approximately one third of these studies (*n* = 22) related to action track 6 with many reporting on climate change abatement initiatives that generate co-benefits for human health such as reduction of disease related to better air quality. One third related to action track 4 (*n* = 20) – with studies commonly evaluating a reduction in pathogen prevalence in live animals or animal products and the resulting reduction of food-borne disease in humans. Twenty four percent of studies related to action track 3 with studies focusing on the control of human disease through disease control measures in the animal reservoir. Eight percent related to action track 2 focussing on preventing zoonotic pandemics as a ‘global public good’ and estimating co-benefits to the human population. Only two studies related to action track 5 and one study related to action track 1. The findings of these studies are presented in Table S10, supplementary materials.

#### 3.3.4 Challenges in conducting economic evaluations of One Health initiatives

The challenges identified in included studies are presented in Table 6.

**Table 6:** Main themes of challenges in conducting economic evaluations identified in included studies (*n* = 97).

Main challenges in conducting One Health economic evaluations	Examples of challenges identified within studies
Limitations in data availability and quality	Limited availability of good quality data was highlighted as a primary issue in many studies. Veterinary costs were often not included in calculations due to absence of reliable data. Such costs included animal vaccination [33], surveillance and diagnostics [15], losses associated with reduced production, decreased reproduction or decreased value of animal products [24,42]. Lack of data for AMR in animals and the environment [43], and role of wildlife hosts in disease transmission were also described as hindering analyses [44]. Likewise, underreporting and/or poor data collection of human health cases was described as a limitation in studies. Available data was not always representative of the country [32,40], and was unable to support the estimation of underreported cases or change in incidence and prevalence following an intervention, leading to underestimation of costs and/or benefits [20,41,[45], [46], [47]]. Reasons for unreliable data reported were that not all cases seek healthcare [20], some diseases studied are not notifiable and therefore, are not recorded or result in asymptomatic infections [48]. If no national data is available, studies use data from similar-context country studies [31,49], secondary data [23,50] or depend largely on expert opinion [37,51,52]. This is especially problematic when trying to make a global investment case [53].
Time horizon: analysing long-term benefits	While it is noted that many effects would be more significant from long-term analyses, as they increase over time [54], it is difficult to accurately predict long term horizons, even with good models [55] and often data on longer-term health impacts are not available [56]. Instead, studies often do not consider long-term effects of interventions [34,57] or if no information is available, costs and benefits are assumed to be constant over the time horizon selected [58].
Metrics used in economic analysis	The lack of standardised measures for effectiveness makes interpretation more challenging and direct comparability or meta-analysis of measures more difficult [29,59]. Other issues described were lack of existing benchmarks for cost-effectiveness ratios of specific interventions, for instance in relation to food safety, to establish whether an intervention is regarded as ‘value for money’ [26]. In contrast, for human health, there are well-established cost-effectiveness thresholds, although there remains some debate about their usefulness [60].
Perspective of economic analysis	The perspective of analysis directly influences the costs and benefits considered. Examples include studies with a public health sector perspective, for instance, food safety studies where industry-borne costs and benefits were not considered [61], or a joint human and animal vaccination program where the costs at the household level to access services was not explicitly included [62], which are important especially for low-resourced populations.
Valuation of wider costs and benefits (e.g., less tangible and non-monetary costs and benefits)	Many categories of expected benefits are not quantified and/or included in studies due to reasons like insufficient data, time, budget [63,64], complexities in identification and quantification of benefits [57] and unknown values [65]. Both health and non-health costs and benefits were described as suffering from these issues. Valuation of non-health benefits like ecosystem benefits or climate benefits were described as being less advanced (than the valuation of health benefits) and difficult to value given they do not have market prices or value [[66], [67], [68], [69]]. Other wider costs and benefits to the economy like (animal/food) trade aspects [22,70], future human and livestock productivity losses or gains from lives saved and effects on tourism revenues [71,72] were common consequences of interventions but not quantified or included in analyses. With reference to health costs and benefits, aspects like reduction in other pathogens as a result of applied interventions [73]; long term costs of illness and death; work absenteeism [19,57] and psychological burden in families of victims were not taken into account [15].
Unequal distribution of costs and benefits between sectors and involved stakeholders	Unequal distribution of costs and benefits between sectors or involved stakeholders may contribute to certain interventions resulting in net economic cost, rather than benefit and consequently contribute to difficulties with implementation [16,[22], [23], [24],^59^,^74^]. With reference to interventions for zoonoses and food safety issues, costs are borne by farmers, producers and the agriculture sector, while the benefits are experienced by public health or the wider economy [16,59,74]. If intervention costs were shared between different sectors based on proportional benefit from control, interventions would more often be cost-effective [[22], [23], [24]]. Alternatively, costs should be compensated from public resources, recognising that the implementation of such interventions are in the interest of the greater society or considered a “public good” [59,74].
Comparison for economic analysis - evidence of the added value (or not) of One Health	Estimating the public health and/or societal costs in the absence of a One Health initiative being implemented was described as challenging or impossible [75,76]. Without this data and a comparator group in the analysis, it is not possible to determine the “added value or not” of One Health.

## 4 Discussion

The findings of this review suggest that there is growing evidence of the added value of One Health and that investments in cross-sectoral health activities can generate acceptable to good economic returns. We found that most included studies reported a positive economic value or return on investment. Most studies demonstrating the added value of One Health focused on evaluating initiatives related to endemic zoonotic diseases and only a small proportion focussed explicitly on evaluating the added value related to using one or more of the four C's of One Health. Studies related to the environment were more frequent when co-benefits were assessed.

### 4.1 Added value of One Health

There is currently limited reporting of the value of the four C's of One Health [1], as evidenced by the minority of studies identified within this review [[14], [15], [16], [17], [18], [19],^62^]. This suggests that most analysts are not investigating the added value of the integrative aspects of One Health. In other words, they are not asking the question whether the core characteristics of One Health are generating the value that is hypothesised by many authors [[14], [15], [16], [17],^19^] (despite the four C's of One Health likely being applied in practice). With the four C's being promoted as central to the achievement of One Health impacts [1], it is important to determine whether it is one or more of the four C's that generate the added value or if it is caused by an extension of the analysis scale and the implicit consideration of externalities.

There is, however, a richer body of evidence that compares cross-sectoral activities with sectoral activities and those that are looking at the generation of co-benefits in other sectors. The latter category report on the cross-sectoral effects of a strategy or intervention that generates some form of improvement – for instance, many studies reported on mitigation or emissions abatement strategies that generate co-benefits for human health [63,66,67,[77], [78], [79], [80], [81], [82]]. Other studies within this category look at the effects of an increase or improvement in an existing strategy with an effect reported in another sector [51,59,61,74,[83], [84], [85], [86], [87], [88], [89]]. Currently, there is insufficient evidence available to assess whether a stronger degree of integration in One Health initiatives correlates with a higher value or return.

In this review, the majority of included studies reported positive economic values, which might be due to publication bias. The review conducted by Falzon et al. reported similar findings [5]. While several studies included in the review had evidence of positive economic outcomes and the added value of using a cross-sectoral approach, many studies showed evidence of positive economic outcomes but failed to demonstrate the added value of One Health. This is likely because most studies are not conceptualised to look at One Health directly and makes it difficult to attribute any additional economic value to the use of a One Health or integrated approach compared to ‘business as usual’. These evaluations are meaningful but should be interpreted with caution when considering the added value of One Health in terms of the economic return of an initiative.

### 4.2 Evidence of economic value of One Health using the One Health JPA action tracks

Action track 1 was considered cross-cutting. Most studies relating to action track 2 demonstrated positive economic outcomes [20,53,65,90,91] but no added value of One Health. Many of these studies referred to One Health initiatives implemented at a global scale using modelling techniques. While these studies are informative, global figures might be too abstract to motivate investment decisions for policy makers and without demonstrated outcomes, it is less clear whether One Health approaches perform as expected [6]. Action track 3 was overrepresented in studies that showed evidence of positive net economic value and the added value of using a One Health approach. These studies are useful to highlight the current evidence base for “good value investments” of One Health initiatives. Most of this literature focused on an anthropocentric economic evaluation of zoonotic disease control (canine-mediated rabies, in particular). Often the costs of these interventions fall on the agriculture sector (or veterinary services) despite benefits being experienced in overall public health or the wider economy [16,59,74]. Costs should be compensated from public resources, recognising that implementing such interventions is in the interest of society as a whole or considered a public good [59,74]. Many of the studies relating to action track 4 did not show positive economic outcomes because the costs of interventions were prohibitive [51,61,70,85,92]. None of the included studies relating to action track 4 explicitly demonstrated the added value of One Health or cross-sectoral (monetary and non-monetary) benefits and most studies showed weak evidence of One Health integration. The perspective for analysis was important for these studies, where a public health sector perspective was often taken, meaning that industry-borne costs and benefits were not considered. Wider costs and benefits specific to food safety like animal and food trade aspects [22,70] or reduction in other pathogens due to the applied intervention [73] were often not able to be accounted for within analyses, resulting in a possible underestimation of benefits.

For action track 5, there was a scarcity of literature economically evaluating AMR from a cross-sectoral perspective. It is quite possible that this gap stems from evaluations conducted through a public health lens and from the limited availability and quality of data on AMR from the animal health and environmental sectors. Almost a quarter of included studies related to action track 6. While the majority of these evaluations showed evidence of positive economic value, they did not include sectoral initiatives as the counterfactual, and it is therefore difficult to assess the economic benefit of using a cross-sectoral approach.

### 4.3 Methods of One Health economic evaluation

Overall, the most common method for conducting economic evaluations of a One Health initiative was CBA, followed by CEA and CUA. CBA aims to evaluate which intervention(s) provides the greater net benefit to society and can include indirect costs and benefits affecting other sectors involved in the initiative, rendering the approach especially beneficial for One Health initiatives [4]. Although CEA measures health benefits in a single unit (DALYs, deaths, YLLs averted etc) and cannot answer the question about how much an intervention will cost and benefit society wholly, it can still help inform decision-making by identifying interventions that offer good value. A bias towards modelling (compared with empirical studies) was observed, which has been noted in previous reviews [5] and is likely due to a combination of data availability, funding, resources, and the complexity of implementation of such interventions. Improved methods are needed to measure and value non-monetary benefits such as improved social and environmental protection as these aspects tend to be excluded from economic evaluations meaning that their value is often not captured in One Health evaluations.

Ultimately, while there is some consistency in the approach used for instance, amongst studies analysing the same One Health topic (e.g., rabies mitigation), there is no overarching methodology or protocol available for the economic evaluation of One Health initiatives. Diversity in economic analysis methodology between studies included in this review resulted in difficulty to compare or combine findings. Reasons for this may include the absence of a standardised framework or lack of quantitative measures to demonstrate the benefits as previously discussed in reviews by Häsler et al. [7] and Baum et al. [6], and the added complexity of evaluations considering their multi-sectoral nature. Naylor et al. reiterates that existing economic evaluation checklists do not offer an appropriate discussion of the health and/or economic impact of cross-sectoral interventions [93] and discussions on complex One Health intervention evaluations are needed for the field [8]. Because One Health is increasingly broad and all-encompassing, reporting standards are used from different disciplines without international agreement on integration for One Health purposes. Going forward, efforts should be put into the development of unified reporting protocol for One Health economic evaluations.

### 4.4 Strengths and limitations

No quality assessment of the included studies was carried out due to resource constraints, which means that results from poorly conducted studies or evaluations that are skewed by biases could have been captured. However, given the limited availability of literature on One Health economics, this approach allowed capturing a wide range of available literature while limiting exclusion of relevant evidence. Google Scholar was not used as part of the search strategy due to limitations in creating a thorough search strategy (its inability to utilise truncation, parentheses etc. compared to bibliographic databases) [94]. The search algorithm is also personalised and therefore not easily replicable. Only studies in English were included due to limited availability of resources, which may have led to language bias and compromised the ability of this review to represent all available literature on the subject. The review utilised the One Health JPA action tracks for the search strategy and subsequent categorisation of data extracted, which helped reveal the areas that receive more or less attention in economic evaluations of One Health. Recent literature has shown that “One Health” is not yet a reliable term for identifying relevant studies for systematic literature reviews [95] and by using the One Health JPA action tracks, many studies were captured that described cross-sectoral effects without using the term One Health. Consequently, several relevant studies were presented covering the health of the environment – an area that is still under-represented in One Health. Some authors may argue that the inclusion of studies that are reporting on cross-sectoral effects with uni-sectoral activities does not meet the more recent One Health definition [1]. However, One Health also conceptualises problems at the system level and looks at interconnections for two or more (health) sectors, which many studies in the third category had done. Also, knowledge on these co-benefits is often important for decision making, even when the One Health evidence is weak.

## 5 Conclusion

Funding and financing for One Health initiatives at the country-level remain a challenge as investments commonly require demonstrated evidence of economic value or return. Decision makers are confronted daily with long lists of competing priorities and economic evaluations of potential investments can be used to decide what initiatives should be prioritised. This review contributes to the growing evidence that can support decision makers with both global and local resource allocation decisions.

We found that evidence gaps exist for many of the action tracks (specifically related to One Health systems strengthening, reducing risks from emerging and re-emerging zoonotic epidemics and pandemics and AMR) indicating where future efforts should be focused. The existing evidence primarily evaluates “traditional” One Health topics such as endemic zoonoses and food safety rather than a broader set of One Health topics and using a systems approach. Some One Health initiatives, in particular canine-mediated rabies control, usually provide a positive economic value and often show the “added value” of One Health through the four C's of One Health. It is evident that health and health economics should be conceptualised in a more holistic sense, incorporating ideas such as animal welfare and ecosystem health, without necessarily being only linked to changes in human health.

The lack of a standardised framework for evaluating the ROI for One Health initiatives has been previously highlighted as a deterrent for the widespread adoption of One Health amongst stakeholders [6]. A practical framework would facilitate assessment of the added value of an integrated approach, generating evidence for the One Health approach to be adopted and endorsed by multiple sectors. This will facilitate advocacy for the added economic value of the four Cs of One Health, to build the evidence base for investment in One Health initiatives.

## Funding

This publication was funded from FAO internal resources (regular programme) and the Bill & Melinda Gates Foundation (MTF/GLO/1040/BMG).

## CRediT authorship contribution statement

**Aashima Auplish:** Conceptualization, Data curation, Formal analysis, Investigation, Methodology, Visualization, Writing – original draft, Project administration. **Eleanor Raj:** Conceptualization, Data curation, Formal analysis, Investigation, Methodology, Visualization, Writing – original draft, Project administration. **Yoeri Booijink:** Data curation, Formal analysis, Methodology, Writing – review & editing. **Katinka de Balogh:** Conceptualization, Methodology, Writing – review & editing. **Marisa Peyre:** Funding acquisition, Methodology, Writing – review & editing, Conceptualization. **Katrin Taylor:** Conceptualization, Funding acquisition, Writing – review & editing. **Keith Sumption:** Conceptualization, Funding acquisition, Writing – review & editing. **Barbara Häsler:** Conceptualization, Data curation, Funding acquisition, Methodology, Project administration, Supervision, Validation, Writing – review & editing.

## Declaration of competing interest

A grant from the Foundation funded AA's, BH's and ER's time. The grant also funded a partnership contract with CIRAD. Project grant code: MTF /GLO/1040/BMG. BH is a member of the One Health Lancet Commission. MP is an expert member of the technical advisory panel of the Pandemic Fund and member of PREZODE initiative secretariat. The authors declare no other competing interests.

## Data Availability

Data will be made available on request.
