# Supplementary material for: Current evidence of the economic value of One Health initiatives: A systematic literature review
Source: One Health. 2024 May 9;18:100755. doi: 10.1016/j.onehlt.2024.100755 (PMC11103946; doi:10.1016/j.onehlt.2024.100755)
Supplement: Supplementary material [file mmc1.docx]

**Supplementary materials**

**Table S1. The detailed search strategies for the databases and sources consulted.**

| ***Database*** | ***Query - with JPA terms*** | ***Number of results*** |
| --- | --- | --- |
| Pubmed | ( "One Health" OR zoono* OR "neglected tropical disease" OR "vector borne" OR "food safety" OR "AMR" OR "antimicrobial resistance" OR "environmental health") AND ("economic evaluation" OR "cost benefit analysis" OR "economic benefit*" OR "cost benefit*" OR "return on investment" OR "economic analys*" OR "economic evaluation" OR "economic assessment" OR "added value" OR "cost effectiveness" OR "cost utility" OR "budget impact analysis" OR "financial analys*" OR "financial evaluation" OR "monetary" OR "investment" OR DALY* OR QALY* OR "disability adjusted life year" OR "disability adjusted life years" OR "quality adjusted life year" OR "quality adjusted life years" OR "opportunity cost" OR "societal benefit*" OR "cost saving" OR "cost avoidance")  **Title/abstract filter applied** | 3116 |
| Scopus | (TITLE-ABS-KEY ( "One Health"  OR  zoono*  OR  "neglected tropical disease"  OR  "vector borne"  OR  "food safety"  OR  "AMR"  OR  "antimicrobial resistance"  OR  "environmental health")) AND  ( TITLE-ABS-KEY ( "economic evaluation"  OR  "cost benefit analysis"  OR  "economic benefit*"  OR  "cost benefit*"  OR  "return on investment"  OR  "economic analys*"  OR  "economic evaluation"  OR  "economic assessment"  OR  "added value"  OR  "cost effectiveness"  OR  "cost utility"  OR  "budget impact analysis"  OR  "financial analys*"  OR  "financial evaluation"  OR  "monetary"  OR  "investment"  OR  daly*  OR  qaly*  OR  "disability adjusted life year"  OR  "disability adjusted life years"  OR  "quality adjusted life year"  OR  "quality adjusted life years"  OR  "opportunity cost"  OR  "societal benefit*"  OR  "cost saving"  OR  "cost avoidance" ) ) | 6119 |
| Web of science | ("One Health" OR zoono* OR "neglected tropical disease" OR "vector borne" OR "food safety" OR "AMR" OR "antimicrobial resistance" OR "environmental health") AND ("economic evaluation" OR "cost benefit analysis" OR "economic benefit*" OR "cost benefit*" OR "return on investment" OR "economic analys*" OR "economic evaluation" OR "economic assessment" OR "added value" OR "cost effectiveness" OR "cost utility" OR "budget impact analysis" OR "financial analys*" OR "financial evaluation" OR "monetary" OR "investment" OR DALY* OR QALY* OR "disability adjusted life year" OR "disability adjusted life years" OR "quality adjusted life year" OR "quality adjusted life years" OR "opportunity cost" OR "societal benefit*" OR "cost saving" OR "cost avoidance")  **‘Topic’ search/ filter applied ie search title, abstract, author keywords, and Keywords Plus only** | 2357 |

**Table S2. Further information about the online survey and workshops conducted.**

| **Activity** | **Description** |
| --- | --- |
| Online survey | The online survey was administered using Survey Monkey between August 12 and October 7, 2022. The survey was circulated through relevant Quadripartite networks and channels and respondents were selected using convenience sampling. Two-hundred and two respondents completed the survey. The purpose of the survey was to gather information from stakeholders globally on the ROI of One Health initiatives to help build the evidence-base and the support the development of a standardised framework for evaluating the ROI for One Health initiatives. Respondents were encouraged to submit any literature (particularly, grey literature) relating to the One Health initiatives they had reported on, which could be included within the review. |
| Workshops | Thirty-five subject matter experts (SMEs) were invited to participate in two virtual workshops in September 2022 and March 2023, to share their knowledge and experience on available theories, concepts, tools and case studies on economic assessments or ROI studies. SMEs were requested to share grey literature that could be included within the review. |

**Table S3. Publication type of studies included within the final analysis (n=97).**

| **Publication type** | **n (%)** |
| --- | --- |
| Book chapter | 1 (1.0) |
| Journal articles | 89 (91.8) |
| Policy brief | 1 (1.0) |
| Report | 6 (6.2) |

**Table S4. Publication format of studies included within the final analysis (n=97).**

| **Publication format** | **n (%)** |
| --- | --- |
| Empirical or case study | 22 (22.3) |
| Empirical or case study/modelling | 37 (38.1) |
| Expert opinion | 1 (1.0) |
| Modelling | 35 (36.1) |
| Other | 2 (2.1) |

**Table S5. Economic evaluation methodology used in studies included within the final analysis (n=97).**

| **Economic evaluation methodology** | **n (%)** |
| --- | --- |
| Cost-benefit analysis | 33 (34.0) |
| Cost-effectiveness analysis | 25 (25.8) |
| Cost-utility analysis | 5 (5.2) |
| Costs and benefits monetised or listed | 30 (30.9) |
| Other | 4 (4.1) |

**Table S6. Socioeconomic and political characteristics of the country where the One Health initiatives from studies included within the final analysis were implemented (n=97).**

|  | **n (%)** |
| --- | --- |
| Global north | 45 (46.4) |
| Global south | 41 (42.3) |
| N/A | 11 (11.3) |

**Table S7. Types of monetary returns used within studies included in the final analysis (n=97)**

| **Type of monetary outcome/return** | **n (%)** |
| --- | --- |
| Cost savings | 17 (17.5) |
| Cost / cases averted | 1 (1.0) |
| Cost / DALYs averted | 2 (2.1) |
| Cost / death averted | 4 (4.1) |
| Average cost-effectiveness ratio | 11 (11.3) |
| Benefit cost ratio | 18 (18.6) |
| Cost utility ratio | 2 (2.1) |
| Incremental cost effectiveness ratio | 11 (11.3) |
| Incremental cost utility ratio | 1 (1.0) |
| Internal rate of return | 1 (1.0) |
| Net benefits | 13 (13.4) |
| Net present value | 8 (8.2) |
| Other | 8 (8.2) |

**Table S8. Perspective used for economic analysis in studies included within final analysis (n=97).**

| **Perspective** | **n (%)** |
| --- | --- |
| Household | 5 (5.2) |
| PH and industry (private sector) | 3 (3.1) |
| Public health | 28 (28.8) |
| Public health and animal health | 10 (10.3) |
| Public health and environmental health | 7 (7.2) |
| Society | 43 (45.4) |

**Table S9. Institution responsible for conducting economic evaluation of One Health initiatives from studies included within final analysis (n=97)**

| **Institution responsible for conducting economic evaluation** | **n (%)** |
| --- | --- |
| Academia | 51 (52.6) |
| Academia / government / non-governmental organisation | 1 (1.0) |
| Academia / private sector | 2 (2.1) |
| Development bank | 5 (5.2) |
| Government | 18 (18.6) |
| Government / academia | 10 (10.3) |
| Government / private sector | 1 (1.0) |
| International organisation | 1 (1.0) |
| International organisation / academia / government | 1 (1.0) |
| Non-governmental organisation | 2 (2.1) |
| Other | 1 (1.0) |
| Private sector | 2 (2.1) |

**Table S10. List of studies demonstrating positive net economic return and but no evidence on the value of using a cross-sectoral vs sectoral comparison.**

​​

| **One Health initiative and level  of implementation** | **Sectors involved in the initiative** | **One Health JPA action track* addressed** | **Economic evaluation method** | **Type and quantification of return on investment** |
| --- | --- | --- | --- | --- |
| One Health systems strengthening to prevent zoonotic disease pandemics implemented at global level [1] | Human health, agriculture/ veterinary services, wildlife health | Action track 1 | CBA | With an investment of US$3.4 billion per year, the annual expected rate of return would be between 44-71% (corresponding to, respectively, half or all mild pandemics being prevented). The global expected benefits are US$30 billion per year. |
| Zoonotic pandemic prevention (utilising strategies like reducing deforestation, cost of surveillance and monitoring in livestock, costs of ending the wild meat trade in China) implemented at the global level [2] | Human health, agriculture/ veterinary services, environmental services. | Action track 2 | Costs and benefits monetised and listed (no methodology specified) | The gross estimated cost of proposed actions in total ranges from $22 to $31 billion per year. Reduced deforestation has the ancillary benefit of around $4 billion per year in social benefits from reduced GHG emissions. In comparison the world may have lost at least $5 trillion in GDP in 2020 and the willingness to pay for the lives lost constitutes many additional trillions. |
| Pandemic prevention (through strategies like strengthening animal and veterinary services, improving on-farm biosecurity, reducing deforestation, improving urban planning) implemented at the global level [3] | Human health, agriculture/veterinary services, wildlife health | Action track 2 | Costs and benefits monetised and listed (no methodology specified) | Prevention costs amount up to $10.3-$11.5 billion annually, compared to $30.1 billion for preparedness |
| Vietnam Avian Influenza Emergency Recovery Project (AIERP), implemented at the national level [4] | Human health, agriculture/ veterinary services | Action track 2 | CBA | The rate of return on the OPI and AIEPED investments in disease control and prevention is 29 percent annually; annual spending $77 million on building and operating veterinary and human public health systems would yield an expected annual benefit of $105 million |
| Primary prevention of zoonotic pandemics (including expanding viral discovery and surveillance; monitoring wildlife hunting and trade and preventing deforestation), implemented at the global level [5] | Human health, agriculture/ veterinary services, wildlife health, environmental services | Action track 2 | Costs and benefits monetised and listed (no methodology specified) | The sum of median cost estimates of primary prevention is approximately $20 billion. The primary pandemic prevention action cost less than 1/20th the value of lives lost each year to emerging viral zoonoses and have substantial cobenefits. |
| National schistosomiasis program in China, implemented at national level [6] | Human health, agriculture/ veterinary services, environmental services | Action track 3 | CBA (ex-ante) | The net benefit-cost ratio was 6.20, which means that this project gained US$ 6.20 for every dollar spent |
| [A raccoon rabies elimination program in Long Island, New Yor](https://www.zotero.org/google-docs/?broken=UCW7sq)k, implemented at the local level [7] | Human health, environmental services, wildlife health | Action track 3 | CBA | The cumulative benefit-cost ratio (benefits each year, divided by the total cost) is projected to reach 1.71 in 2019, inundating every dollar spent on the program will save $1.71 in costs. |
| [Prevention and control strategies for visceral leishmaniasis in Brazil,](https://www.zotero.org/google-docs/?broken=Btl8QC) implemented at national level [8] | Human health, agriculture/ veterinary services | Action track 3 | CEA | To reduce the prevalence in humans and dogs by approximately 70%, the cost ranged from $250,000 and $550.000 for the insecticide-impregnated collar (IIC) and vaccination respectively. Even in the scenario with 40% loss/replacements of IICs, this measure was more advantageous in terms of cost-effectiveness than vaccination. |
| [Canine vaccination and sterilization as rabies control strategy in India](https://www.zotero.org/google-docs/?broken=nYyTo3), implemented at regional level [9] | Human health, agriculture/ veterinary services | Action track 3 | CEA | Annual target of vaccination of 100.00 dogs had an incremental cost-effectiveness ratio (ICER) of $1.064 ($814-$1.447) per DALY, and an annual target of 200.000 dogs had an ICER of $3.964 ($3.255-$4.853). According to WHO criteria these strategies would be considered very cost-effective and cost-effective. |
| Brucellosis control interventions (livestock vaccination, gradual culling through slaughter of seropositive cattle and small ruminants, environmental hygiene and sanitation, and personal protection in humans) in sub-Saharan African countries [10] | Human health, agriculture/ veterinary services, environmental services | Action track 3 | CEA | A combination of livestock vaccination, gradual culling through slaughter, environmental sanitation and personal protection in humans showed an ICER of -0.0638 |
| Zoonotic visceral leishmaniasis control strategies (positive dog elimination, insecticide impregnated dog collar, dog vaccination, dog treatment, and sandfly population control - chemical control and land clearing) [11] | Human health, agriculture/ veterinary services, environmental services | Action track 3 | CEA | Insecticide impregnated dog collar was considered the most efficient and cost-effective among the control strategies with a normalized cost of 0.67, where human patient treatment would have a value of 1.00 with a cost of 397,25 USD per patient. |
| Canine rabies elimination program in the Republic of the Philippines, implemented at the national level [12] | Human health, agriculture/  veterinary services | Action track 3 | Costs and benefits monetised and listed (no methodology specified) | If rabies had been eliminated in 1988, economic benefits would total US$2.5 million in 1989 |
| Cystic echinococcosis control program (in stray dogs, adult sheep and humans) in La Rioja, Spain, implemented at the local level [13] | Human health, agriculture/ veterinary services | Action track 3 | CBA | Accumulated BCR of 1.96 by 2000 (control program evaluated from 1987-2000) |
| Use of oral rabies vaccines for controlling racoon rabies in the US, implemented in multiple provinces or cities [14] | Human health, agriculture/veterinary services | Action track 3 | CBA | NPV of US $3.1 to 2.2 million when the avoidance of increased pet rabies vaccinations is considered as a benefit |
| Large scale oral vaccination program to control raccoon rabies in the US, implemented in multiple provinces or cities [15] | Human health, agriculture/ veterinary services | Action track 3 | CBA | NPV of USD $48 to $422 million (depending on the rates of epizootic spread used in the model and whether cost of animal vaccination was included/excluded) |
| [Deworming foxes to control Echinococcus multilocularis in humans in central Europe,](https://www.zotero.org/google-docs/?broken=hlWhfC) implemented in multiple provinces/cities [16] | Human health, agriculture/ veterinary services | Action track 3 | Costs and benefits monetised and listed (no methodology specified) | Net benefits ranged from €393,105 to -€18,283,796 dependent on the size of the area to implement the program. |
| Genetic vector control, specifically Release of Insects carrying a Dominant Lethal (RIDL) to control dengue in humans [17] | Human health, environmental services | Action track 3 | CEA | This genetic control strategy could eliminate dengue rapidly from a human community, and at lower expense (approximately US$ 2∼30 per case averted) than the direct and indirect costs of disease (mean US$ 86–190 per case of dengue). ICER range from 939 - 2524 USD, which exceed the overall average societal cost per infection (86-190USD) |
| Dog-mediated rabies vaccination campaign in Tanzania implemented in multiple provinces [18] | Human health, agriculture/ veterinary services | Action track 3 | CEA | CER with a 100% effective vaccination = 0.01 (1% of the annual Tanzanian GDP per capita or $7.07 per DALY saved per year) and 1% effective vaccination = 0.75 or $706.56 per DALY saved per year |
| Hydatid disease control program in the Province of Rio Negro, Argentina implemented at the local level [19] | Human health, agriculture/ veterinary services | Action track 3 | Costs and benefits monetised and listed (no methodology specified) | Overall health care costs for 1997 were estimated at US$ 293,215, 77% lower than in 1980 and canine echinococcosis prevalence reduced from 41.5% in 1980 to 4.2% in 1986 and to 2.3% in 1997 (program implemented from 1980-1997) |
| Interventions at farm- (vaccination and meal feeding) and at processor-level (rinsing carcasses at various temperatures with and without sanitiser) to mitigate Salmonella in the United States, implemented at national level [20] | Human health, agriculture/veterinary services | Action track 4 | CBA | BCRs of 0.72 for vaccination of pigs on farms; 0.10 of meal feeding and 1.48-3.55 for various options rinsing carcasses at the processor. |
| [Salmonella control policies for broiler production in Finland](https://www.zotero.org/google-docs/?broken=lPS1DU), Implemented at national level [21] | Human health, agriculture/ veterinary services | Action track 4 | CBA | The median Benefit-Cost ratio for the Finnish Salmonella Control Programme was 4.00 (90% range 0.04-21.25) |
| Regulatory control measures for food safety and Campylobacter in poultry in New Zealand implemented at national level [22] | Human health, agriculture/ veterinary services | Action track 4 | CBA | A benefit-cost ratio of 25.74 was found, with at least $57.4 million saved annually for a total capital investment of $2.014 million and ongoing cost of $0.5 million per annum |
| Campylobacter control strategy consisting of currently available food safety technologies with good consumer acceptance, implemented at regional level [23] | Human health, agriculture/ veterinary services | Action track 4 | CUA | 1.3 million human campylobacteriosis cases could be prevented each year using the suggested strategy, with an annual health benefit of 20 523 QALYs and a net annual cost saving of €17.8 million at the EU-27 level. ICER/GDP per capita ratios by country are in the range of -0.37 to 1.51, except for Finland (5.89) and Sweden (2.80) |
| BSE intervention strategies in the Netherlands, implemented at the national level [24] | Human health, agriculture/ veterinary services | Action track 4 | CEA | The estimated cost-effectiveness of all BSE measures in the Netherlands ranged from 4.3 million euros per life year saved in 2002 to 17.7 million euros in 2005 |
| Control interventions for toxoplasmosis (freezing meat intended for raw or undercooked consumption and improving biosecurity in pig farms) in Netherlands, implemented at the national level [25] | Human health, agriculture/ veterinary services | Action track 4 | CBA | Freezing meat intervention would lead to annual net benefit of €10.6 million and €0.6 million for respectively steak tartare and leg of mutton. Improving biosecurity would result in net costs ranging from €1 million to €2.5 million. From a public health and social perspective, freezing is to be considered. |
| E coli O157:H7 cattle vaccine (used to prevent human infection caused by consuming beef) administered in the United States, implemented at national level [26] | Human health, agriculture/ veterinary services | Action track 4 | CEA | Vaccinating the entire U.S. herd at a cost of between $2.29 and $9.14 (depending on overall effectiveness of the vaccine) would be a cost-effective intervention for preventing E. coli illness in humans. |
| Finnish Salmonella Control Program (FSCP), implemented at the national level [27] | Human health, agriculture/ veterinary services | Action track 4 | Costs and benefits monetised and listed (no methodology specified) | Net benefits of the Salmonella programme for meat production range from €4.793. 953 to €22.983.3120 and egg production €3.884.691 – €11.991.358 €. Reported per household, the estimated benefits were about €3.5 annually |
| Salmonella control program in Denmark, implemented at national level [28] | Human health, agriculture/ veterinary services | Action track 4 | Costs and benefits monetised and listed (no methodology specified) | In 2001, Denmark saved U.S.$25.5 million by controlling Salmonella |
| Interventions to reduce *Campylobacter* levels in the poultry meat value chain in Belgium, implemented at the national level [29] | Human health, agriculture/ veterinary services | Action track 4 | CBA (ex-post) | Decontamination of carcasses with electrolyzed oxidizing water applied in the processing plant was the most efficient (BCR 17.66), followed by the use of lactic acid (BCR 4.06). In addition, phage therapy generated a positive cost-benefit ratio (BCR 2.54) |
| Interventions to control Campylobacter in the poultry meat food supply in New Zealand, implemented at the national level [30] | Human health, agriculture/ veterinary services | Action track 4 | CEA | Using primary processing interventions results in 854-1010 DALYs saved per year |
| Interventions to reduce AMR, implemented at global level [31] | Human health, agriculture/ veterinary services | Action track 5 | Costs and benefits monetised and listed (no methodology specified) | In the optimistic case of low AMR impacts, by 2050, annual global gross domestic product (GDP) would likely fall by 1.1%, relative to a base-case scenario with no AMR effects; the GDP shortfall would exceed $1 trillion annually after 2030. In the high AMR-impact scenario, the world will lose 3.8% of its annual GDP by 2050, with an annual shortfall of $3.4 trillion by 2030. |
| Emission abatement measures, for PM10 and NO2 implemented at regional levels in EU member states, implemented at regional level [32] | Human health, environmental services | Action track 6 | CBA | Net benefit €0.3 million per year, for a scenario where all measures were implemented |
| Installing flue-gas desulfurization units at coal-fired power plants in India, implemented at national level [33] | Human health, environmental services | Action track 6 | CEA | US$5,140 per DALY averted (if FGD units installed at all 72 plants). If ranked by cost-per-life-saved, then 30 most cost-effective plants would have ICER of US$2,600 per DALY averted |
| Implementation of “Green mobility”, “Green Exercise” or “Zero Emission” scenario to improve air quality and human health in urban areas in Austria, implemented at local levels [34] | Human health, environmental services | Action track 6 | Costs and benefits monetised and listed (no methodology specified) | Direct and indirect health costs decrease ranged from €11.8 million to € 19 million per year. GHG emission reduction ranged from 289.680 to 956,5000 tonnes CO2 equivalent. Public investment and operating cost ranged from under €50 million to €500 million per annum. |
| Adaptation of more stringent vehicle emission standards for PM 2.5 in France and Italy, implemented at national levels [35] | Human health, environmental services | Action track 6 | CEA | In France, adopting US emission standards would save €1000 and increase QALYs by 0.04 per capita. In Italy, the stricter standards would save €3000 and increase QALYs by 0.31. |
| Implementation of the National Energy Efficiency Improvement and Energy Conservation Programs (NEEIECP) in Hungary, implemented at the national level [36] | Human health, environmental services | Action track 6 | CBA | BCR by sector (health benefit resulting from reduced emissions vs. the investment in each sector) agriculture = 3; industry = 5; transport = 7; energy sector = 6; HH = 16 and service = 17 |
| Strategies to reduce of PM2.5 concentration to reduce asthma medication use in the United States, implemented at national level [37] | Human health, environmental services | Action track 6 | Costs and benefits monetised and listed (no methodology specified) | A nationwide 1 μg/m3 reduction in particulate matter concentration would generate nearly $350 million annually in economic benefits. |
| Improvement strategies for environmental health in Victorian rivers in Australia, implemented at the local level [38] | Agriculture/ veterinary services, environmental health and wildlife health | Action track 6 | CBA | In total across the state, the environmental health improvements generated by the proposed riparian zone fencing project is estimated at around $12.5 million. Value per household - $32.97 to $40.14 AUD per household |
| Improved management practices for cattle, small ruminants, camels, and poultry in Ethiopia, implemented at the local level [39] | Agriculture/ veterinary services, environment services | Action track 6 | CBA | Deferred- rotation grazing had a positive economic NPV at a 3 percent discount rate over 50 years ($2,069/HH) / active restoration of degraded rangeland NPV of $14,767/HH / fodder cultivation NPV $8,316/HH |
| Fodder production as a low emissions development strategy for the dairy sector in Kenya, implemented at the local level [40] | Agriculture/ veterinary services, environmental services | Action track 6 | CBA | In year 5, investment cost = $7,260,000 and gross profit = $21,402,683 (applies to total # of farmers within 5 counties) |
| Particulate matter related inspection and maintenance programs in Bangkok, Thailand, implemented at local level [41] | Human health, environmental services | Action track 6 | Costs and benefits monetised and listed (no methodology specified) | Annual cost of program is approximately US $147 million; annual total health benefits is US $907.9 to 1483.5 million (from 2008-2015) |
| Retrofitting vehicles with diesel particulate filters to reduce harmful diesel emissions in Mexico City, implemented at local level [42] | Human health, environmental services | Action track 6 | Costs and benefits monetised and listed (no methodology specified) | Diesel oxidation catalysts (DOCs) in newer vehicles costing US$510-780,000 per 1000 vehicles retrofit annually; 0.4 to 3.6 lives saved annually (depending on the type of filter retrofit, age and type of vehicle and average PM reduction due to vehicle retrofit with a catalyzed DPF falls between 85% and 90% when 15 or 30 ppm sulfur diesel was used |
| Acid rain program in the US, implemented at national level [43] | Human health, environmental services | Action track 6 | Costs and benefits monetised and listed (no methodology specified) | Estimated annual net benefits in 2010 US $119 000 (covering human health and ecosystem health) |
| Nitrogen dioxide air pollution control program in Tokyo, Japan, implemented at local level [44] | Human health, environmental services | Action track 6 | CBA (ex-post) | Net benefits of US $6.08 billion in avoided medical costs due to incidence of phlegm and sputum in adults; $775 million in avoided medical costs due to incidence of lower respiratory illness in children; $6.33 billion in avoided costs of lost wages in workers; $833 million in avoided costs of lost wages in mothers caring for their sick children |
| A m[odelled evaluation of long-term health and economic impacts of air pollution under climate change mitigation scenarios in South Korea,](https://www.zotero.org/google-docs/?broken=fxF2LQ) Implemented at national level [45] | Human health, environmental services | Action track 6 | Costs and benefits monetised and listed (no methodology specified) | Monetized co-benefit estimates in 2050 range from 15.62 to 45.16 billion USD, depending on the scenario. Cost of climate change mitigation policy in 2050 has a maximum between 8.50 and 21.15 billion USD. |
| National and local air pollution control measures in Jinan, China, implemented at the local and national level [46] | Human health, environmental services | Action track 6 | Costs and benefits monetised and listed (no methodology specified) | A total of 1.5 billion USD was invested between 2013 and 2017. The total economic benefit associated with health benefits were 317.7 million USD (95%CI: 227.5-458.1) |
| [Implementation of standards for carbon dioxide (CO2) emissions from existing power plants in the US](https://www.zotero.org/google-docs/?broken=6cuGp8), implemented at local level [47] | Human health, environmental services | Action track 6 | Costs and benefits monetised and listed (no methodology specified) | For a highly flexible policy scenario the monetized value of health co-benefits exceed estimated cost for the U.S. By 17 billion USD per year in 2020. Inclusion of social costs of carbon increases benefits from 29 Billion USD to 50 billion USD, with national net benefits of 38 billion USD per year. |
| Climate change mitigation to improve air quality in Asian countries, implemented at the national level [48] | Human health, environmental services | Action track 6 | CBA | Highest potential net benefit of 1.4 trillion USD at country level (in India) |
| Methane (CH4) abatement / mitigation strategies [to decrease surface ozone concentration](https://www.zotero.org/google-docs/?broken=95Wu60), implemented at a global level [49] | Human health, environmental services | Action track 6 | CBA | The marginal cost-effectiveness of 20% methane reduction is estimated to be $420.000 per avoided mortality. If mortalities are valued as $ 1 million each, the benefit is $240 per tonne of Methane, which exceeds the marginal cost of the methane reduction. |

*Action track 1: Enhancing One Health capacities to strengthen health systems
Action track 2: Reducing the risks from emerging and re-emerging zoonotic epidemics and pandemics
Action track 3: Controlling and eliminating endemic zoonotic, neglected tropical and vector-borne diseases
Action track 4: Strengthening the assessment, management and communication of food safety risks

Action track 5: Curbing the silent pandemic of AMR
Action track 6: Integrating the environment into One Health

**References**

[1] W. Bank, *People, pathogens and our planet: the economics of one health*. Washington, 2012.

[2] A. P. Dobso *et al.*, "Ecology and economics for pandemic prevention: Investments to prevent tropical deforestation and to limit wildlife trade will protect against future zoonosis outbreaks," *Science,* Note vol. 369, no. 6502, pp. 379-381, 2020, doi: 10.1126/science.abc3189.

[3] B. World, "Putting Pandemics Behind Us: Investing in One Health to Reduce Risks of Emerging Infectious Diseases," World Bank, Washington, DC, 2022/10// 2022. Accessed: 2023/02/06/16:17:43. [Online]. Available: <https://openknowledge.worldbank.org/handle/10986/38200>

[4] J. Olga, "Implementation completion and results report on the Vietnam avian and human influenza and human pandemic preparedness project financing," The World Bank, ICR00003330, 2014/12/23/ 2014. [Online]. Available: <https://documents1.worldbank.org/curated/en/913201468311659515/pdf/ICR33300P1016000disclosed0120300140.pdf>

[5] A. S. Bernstein *et al.*, "The costs and benefits of primary prevention of zoonotic pandemics," *Science Advances,* vol. 8, no. 5, p. eabl4183, 2022/02/04/ 2022, doi: 10.1126/sciadv.abl4183.

[6] X.-N. Zhou *et al.*, "An economic evaluation of the national schistosomiasis control programme in China from 1992 to 2000," (in eng), *Acta Tropica,* vol. 96, no. 2-3, pp. 255-265, 2005 2005, doi: 10.1016/j.actatropica.2005.07.026.

[7] J. L. Elser, L. L. Bigler, A. M. Anderson, J. L. Maki, D. H. Lein, and S. A. Shwiff, "The Economics of a Successful Raccoon Rabies Elimination Program on Long Island, New York," (in eng), *PLoS Negl Trop Dis,* vol. 10, no. 12, p. e0005062, Dec 2016, doi: 10.1371/journal.pntd.0005062.

[8] A. D. P. Sevá, F. Ferreira, and M. Amaku, "How much does it cost to prevent and control visceral leishmaniasis in Brazil? Comparing different measures in dogs," (in eng), *PLoS One,* vol. 15, no. 7, p. e0236127, 2020, doi: 10.1371/journal.pone.0236127.

[9] M. C. Fitzpatrick *et al.*, "One Health approach to cost-effective rabies control in India," (in eng), *Proc Natl Acad Sci U S A,* vol. 113, no. 51, pp. 14574-14581, Dec 20 2016, doi: 10.1073/pnas.1604975113.

[10] N. Nyerere, L. S. Luboobi, S. C. Mpeshe, and G. M. Shirima, "Optimal Control Strategies for the Infectiology of Brucellosis," *International Journal of Mathematics and Mathematical Sciences,* vol. 2020, May 2020, Art no. 1214391, doi: 10.1155/2020/1214391.

[11] H. J. Shimozako, J. Wu, and E. Massad, "The Preventive Control of Zoonotic Visceral Leishmaniasis: Efficacy and Economic Evaluation," (in eng), *Computational and Mathematical Methods in Medicine,* vol. 2017, p. 4797051, 2017 2017, doi: 10.1155/2017/4797051.

[12] D. B. Fishbein *et al.*, "Rabies control in the Republic of the Philippines: benefits and costs of elimination," (in en), *Vaccine,* vol. 9, no. 8, pp. 581-587, 1991/08/01/ 1991, doi: 10.1016/0264-410X(91)90246-3.

[13] S. Jiménez, A. Pérez, H. Gil, P. Schantz, E. Ramalle, and R. Juste, "Progress in control of cystic echinococcosis in La Rioja, Spain: decline in infection prevalences in human and animal hosts and economic costs and benefits," (in eng), *Acta Tropica,* vol. 83, no. 3, pp. 213-221, 2002/09// 2002, doi: 10.1016/s0001-706x(02)00091-8.

[14] M. I. Meltzer, "Assessing the costs and benefits of an oral vaccine for raccoon rabies: a possible model," (in eng), *Emerging Infectious Diseases,* vol. 2, no. 4, pp. 343-349, 1996 1996, doi: 10.3201/eid0204.960411.

[15] P. Kemere, M. Liddel, P. Evangelou, D. Slate, and S. Osmek, "Economic analysis of a large scale oral vaccination program to control raccoon rabies," *Human Conflicts with wildlife: Economic Considerations,* 2000/08/01/ 2000.

[16] D. Hegglin and P. Deplazes, "Control of Echinococcus multilocularis: strategies, feasibility and cost-benefit analyses," (in eng), *Int J Parasitol,* vol. 43, no. 5, pp. 327-37, Apr 2013, doi: 10.1016/j.ijpara.2012.11.013.

[17] N. Alphey, L. Alphey, and M. B. Bonsall, "A model framework to estimate impact and cost of genetics-based sterile insect methods for dengue vector control," (in eng), *PLoS One,* vol. 6, no. 10, p. e25384, 2011, doi: 10.1371/journal.pone.0025384.

[18] F. O. Fasina *et al.*, "Where and when to vaccinate? Interdisciplinary design and evaluation of the 2018 Tanzanian anti-rabies campaign," (in eng), *Int J Infect Dis,* vol. 95, pp. 352-360, Jun 2020, doi: 10.1016/j.ijid.2020.03.037.

[19] E. Larrieu *et al.*, "Evaluation of the losses produced by hydatidosis and cost/benefit analysis of different strategic interventions of control in the Province of Rio Negro, Argentina," (in English), *Boletín Chileno de Parasitología,* vol. 55, no. 1/2, pp. 8-13, 2000 2000.

[20] G. Y. Miller, X. Liu, P. E. McNamara, and D. A. Barber, "Influence of Salmonella in pigs preharvest and during pork processing on human health costs and risks from pork," (in eng), *J Food Prot,* vol. 68, no. 9, pp. 1788-98, Sep 2005, doi: 10.4315/0362-028x-68.9.1788.

[21] S. Kangas, T. Lyytikäinen, J. Peltola, J. Ranta, and R. Maijala, "Costs of two alternative Salmonella control policies in Finnish broiler production," (in eng), *Acta Vet Scand,* vol. 49, no. 1, p. 35, Dec 4 2007, doi: 10.1186/1751-0147-49-35.

[22] D. Gail, "Determining the health benefits of poultry industry compliance measures: the case of campylobacteriosis regulation in New Zealand," *New Zealand Medical Journal,* vol. 117, no. 1391, 2014 2014.

[23] J. G. Pitter, Z. Vokó, Á. Józwiak, and A. Berkics, "Campylobacter control measures in indoor broiler chicken: critical re-assessment of cost-utility and putative barriers to implementation," (in eng), *Epidemiol Infect,* vol. 146, no. 11, pp. 1433-1444, Aug 2018, doi: 10.1017/s0950268818001528.

[24] A. Benedictus, H. Hogeveen, and B. R. Berends, "The price of the precautionary principle: Cost-effectiveness of BSE intervention strategies in the Netherlands," *Preventive Veterinary Medicine,* vol. 89, no. 3-4, pp. 212-222, Jun 2009, doi: 10.1016/j.prevetmed.2009.03.001.

[25] A. W. M. Suijkerbuijk *et al.*, "A social cost-benefit analysis of two One Health interventions to prevent toxoplasmosis," (in eng), *PLoS One,* vol. 14, no. 5, p. e0216615, 2019, doi: 10.1371/journal.pone.0216615.

[26] J. Withee, M. Williams, T. Disney, W. Schlosser, N. Bauer, and E. Ebel, "Streamlined analysis for evaluating the use of preharvest interventions intended to prevent Escherichia coli O157:H7 illness in humans," (in eng), *Foodborne Pathog Dis,* vol. 6, no. 7, pp. 817-25, Sep 2009, doi: 10.1089/fpd.2008.0255.

[27] R. Maijala and J. Peltola, "Finnish Salmonella Control Program -- Efficiency and Viability in Food Safety Promotion," AgEcon Search, 2002 2002. Accessed: 2023/02/06/16:25:11. [Online]. Available: <http://purl.umn.edu/24793>

[28] H. C. Wegener *et al.*, "Salmonella control programs in Denmark," (in eng), *Emerging Infectious Diseases,* vol. 9, no. 7, pp. 774-780, 2003/07// 2003, doi: 10.3201/eid0907.030024.

[29] X. Gellynck, W. Messens, D. Halet, K. Grijspeerdt, E. Hartnett, and J. Viaene, "Economics of reducing Campylobacter at different levels within the Belgian poultry meat chain," (in eng), *Journal of Food Protection,* vol. 71, no. 3, pp. 479-485, 2008/03// 2008, doi: 10.4315/0362-028x-71.3.479.

[30] R. J. Lake, B. J. Horn, A. H. Dunn, R. Parris, F. T. Green, and D. C. McNickle, "Cost-effectiveness of interventions to control Campylobacter in the New Zealand poultry meat food supply," (in eng), *Journal of Food Protection,* vol. 76, no. 7, pp. 1161-1167, 2013/07// 2013, doi: 10.4315/0362-028X.JFP-12-481.

[31] W. Bank, *Drug-resistant infections: a threat to our economic future*. World Bank, 2017.

[32] A. I. Miranda *et al.*, "A cost-efficiency and health benefit approach to improve urban air quality," (in en), *Science of The Total Environment,* vol. 569-570, pp. 342-351, 2016/11/01/ 2016, doi: 10.1016/j.scitotenv.2016.06.102.

[33] M. L. Cropper, S. Guttikunda, P. Jawahar, K. Malik, and I. Partridge, "Costs and Benefits of Installing Flue-Gas Desulfurization Units at Coal-Fired Power Plants in India," in *Injury Prevention and Environmental Health*, C. N. Mock, R. Nugent, O. Kobusingye, and K. R. Smith Eds. Washington (DC): The International Bank for Reconstruction and Development / The World Bank

© 2017 International Bank for Reconstruction and Development / The World Bank., 2017.

[34] B. Wolkinger *et al.*, "Evaluating Health Co-Benefits of Climate Change Mitigation in Urban Mobility," (in eng), *Int J Environ Res Public Health,* vol. 15, no. 5, Apr 28 2018, doi: 10.3390/ijerph15050880.

[35] S. Kim, C. Xiao, I. Platt, Z. Zafari, M. Bellanger, and P. Muennig, "Health and economic consequences of applying the United States' PM(2.5) automobile emission standards to other nations: a case study of France and Italy," (in eng), *Public Health,* vol. 183, pp. 81-87, Jun 2020, doi: 10.1016/j.puhe.2020.04.024.

[36] K. Aunan, G. Pátzay, H. Asbjørn Aaheim, and H. Martin Seip, "Health and environmental benefits from air pollution reductions in Hungary," (in eng), *Sci Total Environ,* vol. 212, no. 2-3, pp. 245-68, Apr 8 1998, doi: 10.1016/s0048-9697(98)00002-3.

[37] A. M. Williams, D. J. Phaneuf, M. A. Barrett, and J. G. Su, "Short-term impact of PM(2.5) on contemporaneous asthma medication use: Behavior and the value of pollution reductions," (in eng), *Proc Natl Acad Sci U S A,* vol. 116, no. 12, pp. 5246-5253, Mar 19 2019, doi: 10.1073/pnas.1805647115.

[38] J. Bennett, R. Dumsday, G. Howell, C. Lloyd, N. Sturgess, and L. Van Raalte, "The economic value of improved environmental health in Victorian rivers," *Australasian Journal of Environmental Management,* vol. 15, no. 3, pp. 138-148, Sep 2008.

[39] S. Ng’ang’a, S. Gordon, M. Chris, A. Sintayehu, G. Evan, and H. Eric, "Cost Benefit Analysis of Improved Livestock Management Practices in the Oromia Lowlands of Ethiopia," USAID, 2020/10/14/ 2020. Accessed: 2023/02/06/16:41:38. [Online]. Available: <https://www.climatelinks.org/resources/cost-benefit-analysis-improved-livestock-management-practices-oromia-lowlands-ethiopia>

[40] J. Kashangaki and P. J. Ericksen, "Cost–benefit analysis of fodder production as a low emissions development strategy for the Kenyan dairy sector," International Livestock Research Institute, Report 2018/07/15/ 2018. Accessed: 2023/02/06/16:42:27. [Online]. Available: <https://cgspace.cgiar.org/handle/10568/97426>

[41] Y. Li and D. J. Crawford-Brown, "Assessing the co-benefits of greenhouse gas reduction: health benefits of particulate matter related inspection and maintenance programs in Bangkok, Thailand," (in eng), *The Science of the Total Environment,* vol. 409, no. 10, pp. 1774-1785, 2011/04/15/ 2011, doi: 10.1016/j.scitotenv.2011.01.051.

[42] G. Stevens, A. Wilson, and J. K. Hammitt, "A benefit-cost analysis of retrofitting diesel vehicles with particulate filters in the Mexico City metropolitan area," (in eng), *Risk Analysis: An Official Publication of the Society for Risk Analysis,* vol. 25, no. 4, pp. 883-899, 2005/08// 2005, doi: 10.1111/j.1539-6924.2005.00650.x.

[43] L. G. Chestnut and D. M. Mills, "A fresh look at the benefits and costs of the US acid rain program," (in eng), *Journal of Environmental Management,* vol. 77, no. 3, pp. 252-266, 2005/11// 2005, doi: 10.1016/j.jenvman.2005.05.014.

[44] A. S. Voorhees, S. Araki, R. Sakai, and H. Sato, "An ex post cost-benefit analysis of the nitrogen dioxide air pollution control program in Tokyo," (in eng), *Journal of the Air & Waste Management Association (1995),* vol. 50, no. 3, pp. 391-410, 2000/03// 2000, doi: 10.1080/10473289.2000.10464027.

[45] S. E. Kim *et al.*, "Air quality co-benefits from climate mitigation for human health in South Korea," (in eng), *Environ Int,* vol. 136, p. 105507, Mar 2020, doi: 10.1016/j.envint.2020.105507.

[46] L. Cui, J. Zhou, X. Peng, S. Ruan, and Y. Zhang, "Analyses of air pollution control measures and co-benefits in the heavily air-polluted Jinan city of China, 2013–2017," *Scientific Reports,* Article vol. 10, no. 1, 2020, Art no. 5423, doi: 10.1038/s41598-020-62475-0.

[47] J. J. Buonocore, K. F. Lambert, D. Burtraw, S. Sekar, and C. T. Driscoll, "An Analysis of Costs and Health Co-Benefits for a U.S. Power Plant Carbon Standard," (in en), *PLOS ONE,* vol. 11, no. 6, p. e0156308, 2016/06/07/ 2016, doi: 10.1371/journal.pone.0156308.

[48] Y. Xie *et al.*, "Co-benefits of climate mitigation on air quality and human health in Asian countries," (in eng), *Environ Int,* vol. 119, pp. 309-318, Oct 2018, doi: 10.1016/j.envint.2018.07.008.

[49] J. J. West, A. M. Fiore, L. W. Horowitz, and D. L. Mauzerall, "Global health benefits of mitigating ozone pollution with methane emission controls," (in eng), *Proc Natl Acad Sci U S A,* vol. 103, no. 11, pp. 3988-93, Mar 14 2006, doi: 10.1073/pnas.0600201103.
